# Oncologic multivisceral resections involving the pancreas: Short-term outcomes and risk-analysis

**DOI:** 10.1097/JS9.0000000000003731

**Published:** 2026-03-11

**Authors:** Artur Rebelo, Bodil Andersson, Samik Kumar Bandyopadhyay, Paulina Bereza-Carlson, Frederik Berrevoet, Bergthor Björnsson, Stefan Bouwense, Florian Bösch, Markus Büchler, Nikolaos Chatzizacharias, Laurent Coubeau, Marie Crede, Cristine B. Pathirannehalage Don, Pieter Dries, Matthäus Felsenstein, Isabella Frigerio, Alessandro Giardino, Tim Glowka, Parsa Hadesi, Vera Hartman, Karin Johansen, Marie Klein, Johannes Klose, Karl Knipper, Carl-Stephan Leonhardt, Martin Loos, Thomas Malinka, Giovanni Marchegiani, Christopher Månsson, Riccardo Pellegrini, Giampaolo Perri, Lh Poelsler, Geert Roeyen, Michael Rousek, Pablo Sancho, Stina Schild-Suhren, Thomas Schmidt, Leyre Serrablo, Alejandro Serrablo, Andrew Malvern Smith, Gregor A. Stavrou, Oliver Strobel, Alexandra Strobel, Ignazio Tarantino, Jozef Urdzik, Tim Vilz, Roberta Vella, Johanna Wennerblom, Patricia Wyzlic, Jörg Kleeff

**Affiliations:** a Department of Visceral, Vascular and Endocrine Surgery, University Hospital Halle (Saale), Martin-Luther-University Halle-Wittenberg, Halle, Germany; b Department of Clinical Sciences, Lund University, Lund, Sweden; c Division of Hepatobiliary and Pancreatic Surgery, Skåne University Hospital, Lund, Sweden; d Manchester Royal Infirmary, UK; e Department of General and HPB Surgery, Liver Transplantation University Hospital Gent, Gent, Belgium; f Department of Surgery in Linköping and Department of Biomedical and Clinical Sciences, Linköping University, Linköping, Sweden; g Department of Surgery, Maastricht University Medical Centre, Gent, Netherlands; h NUTRIM School of Nutrition and Translational Research in Metabolism, Faculty of Health, Medicine and Life Sciences, Maastricht University, Gent, Netherlands; i Department of General, Visceral and Pediatric Surgery, University Medical Center Göttingen, Göttingen, Germany; j Botton-Champalimaud Pancreatic Cancer Center, Lisbon, Portugal; k HPB and Liver Transplant Unit, Queen Elizabeth Hospital, Birmingham, UK; l Institute of Clinical Sciences, University of Birmingham, Birmingham, UK; m Department of Abdominal Surgery, Transplantation at Cliniques Universitaires Saint-Luc, Brussels, Belgium; n Department of Surgery, Charité – Universitätsmedizin Berlin, Berlin, Germany; o HPB Unit Peschiera del Garda, Italy; p Collegium Medicum, SAN University, Lodz, Poland; q Department of General and Visceral Surgery, Charité - Universitätsmedizin Berlin, corporate member of Freie Universität Berlin und Humboldt-Universität zu Berlin, Campus Benjamin Franklin, Berlin, Germany; r Department of Surgery, Institute of Clinical Sciences, Sahlgrenska Academy, University of Gothenburg, Gothenburg, Swedens Antwerp Belgiumu Cologne Germanyv Vienna, Austriaw Heidelberg, Germany Uppsale, Sweden; s Department of HPB, Endocrine and Transplantation Surgery, Antwerp University Hospital, Belgium; t Department of General, Visceral, Endocrine, and Transplant Surgery, Cantonal Hospital of St. Gallen, Switzerland; u Department of General, Visceral, Thoracic and Transplantation Surgery, University Hospital of Cologne, Germany; v Department of General Surgery, Division of Visceral Surgery, Medical University of Vienna, Austria; w Department of General, Visceral and Transplantation Surgery, Heidelberg University Hospital, Germany; x Department of Surgical, Oncological and Gastroenterological Sciences, University of Padua, Padua, Italy; y Department of Surgical Sciences, Uppsala University, Sweden; z Department of Visceral, Transplant and Thoracic Surgery, Univeristy Innsbruck, Innsbruck, Austria; aa Department of Surgery, Second Faculty of Medicine of Charles University and Military University Hospital, Prague, Czech Republic; bb HPB Surgical Division, Miguel Servet University Hospital, Zaragoza, Spain; cc St James University Hospital, Leeds, UK; dd Department of General, Visceral and Thoracic Surgery, Surgical Oncology, Klinikum Saarbruecken, Saarbruecken, Germany; ee Interdisciplinary Center for Health Sciences, Institute of Medical Epidemiology, Biostatistics, and Informatics, Medical Faculty of Martin Luther University Halle-Wittenberg, Halle /Saale, Germany; ff Institute of Health, Midwifery and Nursing Science, Medical Faculty of Martin Luther University Halle-Wittenberg, University Medicine Halle, Halle /Saale, Germany; gg Dept of Surgery, Sahlgrenska University Hospital, Region Västra Götaland, Gothenburg, Sweden

**Keywords:** multivisceral resection, oncological resections, outcomes, pancreatic malignancies, risk factors

## Abstract

**Objective:**

To evaluate short-term outcomes and identify predictors of morbidity and mortality following multivisceral oncologic resections involving the pancreas.

**Summary background data:**

Multivisceral resections including the pancreas are required for locally advanced abdominal malignancies but are associated with considerable perioperative risk. While smaller series suggest acceptable outcomes in selected patients, large-scale international data are lacking to guide surgical decision-making and risk stratification.

**Methods:**

This was a retrospective cohort study of 1283 patients from 31 international centers who underwent multivisceral oncologic resections involving the pancreas. Patient demographics, tumor characteristics, operative details, and 90-day postoperative outcomes were analyzed.

**Results:**

The cohort had a mean age of 64.7 years, and 54.7% were male. Distal pancreatectomy was the most frequent procedure (60.5%), and R0 resection was achieved in 60.9% of cases. The 90-day mortality was 6.9%, highest in patients with gastric adenocarcinoma (16.7%). Major complications (Clavien–Dindo grade III–V) occurred in 34.4% of patients. Higher ASA classification and open surgical approach were independently associated with increased morbidity and mortality. Prolonged operative time was associated with morbidity only. Female gender and treatment at high-volume centers were protective. In patients with pancreatic tumors, resection involving the colon (OR: 1.78, *P* < 0.001), stomach (OR: 1.33, *P* = 0.042), or three or more organs (OR: 1.75, *P* = 0.006) significantly increased complication rates.

**Conclusions:**

Multivisceral resections involving the pancreas are associated with relevant perioperative risk. Optimizing patient selection, favoring minimally invasive techniques when feasible in selected patients, and centralizing care to high-volume centers may help improve outcomes for these complex surgical procedures.

## Introduction

Complete resection stands as the principal curative recourse for non-metastatic solid malignancies. However, in locally advanced stages with involvement of adjacent organs or structures, the mere excision of the tumor’s origin may prove insufficient. In such scenarios, the complete tumor removal necessitates a multivisceral resection^[1]^.

Among advanced abdominal and retroperitoneal tumors such as sarcomas, colon cancer, pancreatic cancer, and gastric cancer, the organs most frequently subjected to resection encompass the colon, stomach, and the pancreas^[2–7]^. While isolated pancreatic operations are acknowledged as intricate interventions bearing considerable risks, including relevant mortality and morbidity, a noteworthy proportion of patients, roughly one-third, undergo pancreatic resection as part of a broader multivisceral resection^[8]^.

The adoption of such aggressive resections can potentially enhance the prospect of cure. However, due to the additional surgical trauma, these interventions also introduce supplementary hazards that can compromise outcomes and might diminish survival^[9–15]^.

The evidence concerning the impact of multivisceral oncological resections involving the across varied tumor entities is characterized by heterogeneity. This study represents a large-scale, international, multicenter investigation designed to overcome the limitations of small patient cohorts in prior research.

The primary objective of this study is to evaluate the short-term outcomes of multivisceral oncologic resections involving the pancreas in a large international cohort, assessing perioperative morbidity, mortality, and potential predictors of surgical complications and postoperative mortality. This study therefore aims to provide insights into patient selection, risk stratification, and surgical decision-making to optimize clinical outcomes.

## Methods

### Study design and setting

This study is a retrospective, multicenter, international cohort study. Data were collected from surgical centers worldwide, focusing on patients who underwent these procedures between 1 January 2010 and 31 December 2022. Data were prospectively collected and entered by local investigators into a centralized REDCap database, allowing standardized data acquisition across institutions. The study followed local ethical and regulatory standards, including anonymized data collection when patient consent is not required. Approval from the Ethics Committee was obtained. Additionally, all other participating centers obtained local ethical approval in accordance with national and institutional guidelines. The study protocol was reviewed, approved, and supported by the European-African Hepato-Pancreato-Biliary Association (E-AHPBA). The work has been reported in line with the STROCSS (Strengthening the reporting of cohort, cross-sectional and case-control studies in surgery) criteria^[16]^.

**HIGHLIGHTS**

- This international multicenter cohort study included 1283 patients from 31 high-volume centers undergoing multivisceral oncologic pancreatic resections.
- The overall 90-day mortality rate was 6.9%, with severe postoperative complications occurring in 34.4% of cases.
- Higher ASA classification and open surgical approach were independent predictors of increased morbidity and mortality.
- Multivisceral resections involving the colon, stomach, or ≥3 organs significantly increased complication rates in patients with pancreatic tumors.

### Participants

#### Inclusion criteria

The study included patients who underwent elective multivisceral oncological resections involving the pancreas. Only cases performed with curative intent for non-metastatic malignancies requiring *en bloc* resection of adjacent organs were considered eligible.

#### Exclusion criteria

Patients were excluded if they underwent isolated pancreatic resections or resections performed for traumatic lesions or other non-oncologic indications. Additionally, cases involving palliative resections without curative intent, revision pancreatectomies, or pancreatic metastases from other primary tumors were not included in the analysis.

### Intervention

The study investigates patients who underwent multivisceral oncologic resections, defined as pancreatic resection (total pancreatectomy, distal pancreatectomy, or partial pancreatoduodenectomy) combined with the resection of at least one additional organ, including: colon, stomach, adrenal gland, liver, kidney, small intestine and spleen. Portal vein or arterial resections, jejunal resection, or partial stomach resection performed as part of a standard pancreatoduodenectomy, as well as splenectomy conducted during a standard distal pancreatectomy, were not classified as multivisceral resections. Resections of premalignant pancreatic lesions were also included.

### Outcomes

The primary outcome of this study was 90-day postoperative mortality. Secondary outcomes included perioperative morbidity, which was classified according to the Clavien–Dindo classification^[17]^, as well as surgical complications such as pancreatic fistula, postoperative bleeding, and delayed gastric emptying, defined according to the ISGPS criteria^[18–20]^. Reoperation rates and intraoperative adverse events were assessed using the Satava classification^[21]^. Resection margin status (R0, R1, and R2) was determined based on the definitions of the Royal College of Pathologists^[22]^. Additionally, the length of stay in the intensive care unit (ICU, in days) and the total hospital length of stay (in days) was evaluated.

### Statistical analysis

Demographic and operative characteristics were summarized using appropriate descriptive summary measures and were compared between the subgroups using Chi-squared tests for categorical variables and ANOVA for continuous variables. Depending on the outcome scale, logistic or ordinal regressions were used to examine univariable associations between risk factors and mortality and morbidity. Subsequently, a multivariable logistic and ordinal regression model was examined comprising risk factors selected by a backwards selection procedure. A two-sided 5% significance level was assumed; however, all results are of exploratory nature. Missing data were assumed to be missing completely at random (MCAR). Therefore, complete case analyses were performed. Additionally, we provided the proportion of missingness in each variable. Kaplan–Meier analyses were not performed, as the primary endpoint of this study was 90-day postoperative mortality rather than long-term survival. RStudio (version 4.4.2) was used for data analysis.

## Results

**Figure 1.**
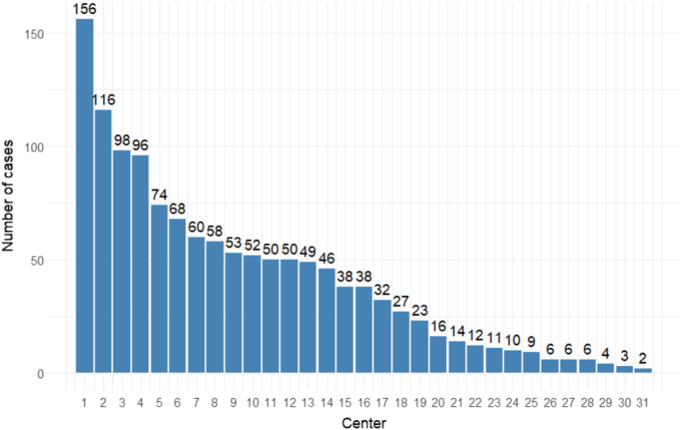
Number of patients included stratified by center.

In total 1283 patients from 31 centers undergoing oncologic multivisceral pancreatic resections were included (Fig. 1). Of 1283 patients, 51.5% had pancreatic adenocarcinoma (*N* = 661), 12.3% had pancreatic NET (*N* = 158), 5.5% had cystic pancreatic lesions (*N* = 71), 3.4% had liposarcoma (*N* = 43), 3.3% had gastric adenocarcinoma (*N* = 42), 3.8% had cholangiocarcinoma (*N* = 49), and 20.2% were classified as other malignancies (*N* = 259), which included leiomyosarcoma (*N* = 11), hepatocellular carcinoma (HCC; *N* = 13), gastrointestinal stromal tumors (GISTs; *N* = 22), and non-pancreatic NET (*N* = 11).The mean age of the overall cohort was 64.7 years (SD: 21.6). The mean BMI was 24.9 kg/m^2^ (SD: 5.21). The Charlson Comorbidity Index (CCI) ^[23]^ had a mean of 0.928 (SD: 1.20) overall. ASA classification ^[24]^ showed most patients were ASA II (43.5%) or III (33.7%). ECOG performance status showed most patients were ECOG 1 (43.4%). Higher ECOG scores were uncommon. Lymph node involvement was observed in 31.5% overall. Distant metastasis (M1) was present in 21.8% of cases, highest in NETs (45.6%) and lowest in cystic pancreatic neoplasia (2.8%; *P* < 0.001). R0 resection was achieved in 60.9% of cases. The highest rates were seen in cholangiocarcinoma (83.7%) and gastric adenocarcinoma (73.8%). R1/R2 resections were more common in liposarcoma (41.9%). Neoadjuvant chemotherapy was given in 24.3% overall, most frequently in gastric (33.3%) and pancreatic adenocarcinoma (30.4%), and least in cholangiocarcinoma (6.1%) and cystic pancreatic neoplasia (4.2%; *P* < 0.001). Adjuvant chemotherapy was administered in 42.4% of cases, with the highest proportions in cholangiocarcinoma (61.2%) and pancreatic adenocarcinoma (50.7%), and the lowest in NETs (24.7%) and cystic pancreatic neoplasia (9.9%; *P* < 0.001). Regarding type of pancreatic resection, distal pancreatectomy was most common (60.5%), particularly in NETs (69.6%) and gastric cancer (71.4%). Total pancreatectomy was least frequent (9.6%; *P* < 0.001). The mean duration of surgery for the overall cohort was 300 minutes (SD: 132). Minimally invasive surgery was performed in 8.9% overall, most commonly in cystic pancreatic neoplasia (23.9%) and NETs (12.7%), and not performed at all in gastric or cholangiocarcinoma (*P* < 0.001). Concerning the additional organ resected, colon (30.9%), stomach (27.6%), and adrenal gland (25.8%) were more frequent. Kidney, small intestine, and other structures were less frequently resected (*P* = 0.005). The high rates of splenectomy can be attributed to the performance of total or distal pancreatectomies with splenectomy, during which additional organs were also resected. Most patients had one (53.0%) or two (30.1%) additional organs resected. (Table 1)

**Table 1:** Patient characteristics for the overall cohort stratified by tumor entity.

	Pancreatic adenocarcinoma (*N* = 661)	Pancreatic NET (*N* = 158)	Cystic Pancreatic Lesions (*N* = 71)	Liposarcoma (*N* = 43)	Gastric adenocarcinoma (*N* = 42)	Cholangiocarcinoma (*N* = 49)	Other^a^ (*N* = 259)	Overall (*N* = 1283)	*P*-Value
Age (years)									0.008
Mean (SD)	66.7 (27.2)	59.8 (12.6)	62.6 (15.5)	65.6 (10.8)	61.7 (13.8)	66.1 (11.8)	62.9 (12.9)	64.7 (21.6)	
Missing	6 (0.9%)	1 (0.6%)	1 (1.4%)	0 (0%)	1 (2.4%)	0 (0%)	2 (0.8%)	11 (0.9%)	
Gender									
Male	368 (55.7%)	79 (50.0%)	28 (39.4%)	23 (53.5%)	27 (64.3%)	28 (57.1%)	149 (57.5%)	702 (54.7%)	
Female	292 (44.2%)	79 (50.0%)	43 (60.6%)	20 (46.5%)	15 (35.7%)	21 (42.9%)	110 (42.5%)	580 (45.2%)	
Missing	1 (0.2%)	0 (0%)	0 (0%)	0 (0%)	0 (0%)	0 (0%)	0 (0%)	1 (0.1%)	
BMI (kg/m^2^)									0.672
Mean (SD)	24.6 (4.63)	27.2 (5.89)	26.1 (5.33)	26.7 (4.94)	23.5 (5.64)	26.3 (5.20)	25.5 (7.84)	24.9 (5.21)	
Missing	74 (11.2%)	19 (12.0%)	5 (7.0%)	5 (11.6%)	5 (11.9%)	1 (2.0%)	33 (12.7%)	141 (11.0%)	
CCI (points)									0.060
Mean (SD)	0.908 (1.15)	1.19 (1.37)	0.714 (0.967)	0.721 (1.32)	0.690 (0.975)	0.833 (1.08)	0.961 (1.26)	0.928 (1.20)	
Missing	126 (19.1%)	15 (9.5%)	15 (21.1%)	0 (0%)	0 (0%)	1 (2.0%)	5 (1.9%)	162 (12.6%)	
ASA									0.016
1	75 (11.3%)	28 (17.7%)	16 (22.5%)	2 (4.7%)	5 (11.9%)	6 (12.2%)	47 (18.1%)	179 (14.0%)	
2	293 (44.3%)	78 (49.4%)	33 (46.5%)	20 (46.5%)	19 (45.2%)	15 (30.6%)	100 (38.6%)	558 (43.5%)	
3	237 (35.9%)	41 (25.9%)	18 (25.4%)	20 (46.5%)	15 (35.7%)	18 (36.7%)	84 (32.4%)	433 (33.7%)	
4	11 (1.7%)	1 (0.6%)	0 (0%)	0 (0%)	0 (0%)	1 (2.0%)	8 (3.1%)	21 (1.6%)	
Missing	45 (6.8%)	10 (6.3%)	4 (5.6%)	1 (2.3%)	3 (7.1%)	9 (18.4%)	20 (7.7%)	92 (7.2%)	
ECOG									0.218
1	285 (43.1%)	78 (49.4%)	37 (52.1%)	17 (39.5%)	24 (57.1%)	13 (26.5%)	103 (39.8%)	557 (43.4%)	
2	218 (33.0%)	44 (27.8%)	17 (23.9%)	18 (41.9%)	13 (31.0%)	7 (14.3%)	68 (26.3%)	385 (30.0%)	
3	36 (5.4%)	6 (3.8%)	9 (12.7%)	2 (4.7%)	0 (0%)	1 (2.0%)	17 (6.6%)	71 (5.5%)	
4	10 (1.5%)	1 (0.6%)	1 (1.4%)	0 (0%)	0 (0%)	1 (2.0%)	6 (2.3%)	19 (1.5%)	
Missing	112 (16.9%)	29 (18.4%)	7 (9.9%)	6 (14.0%)	5 (11.9%)	27 (55.1%)	65 (25.1%)	251 (19.6%)	
T									<0.001
1	58 (8.8%)	30 (19.0%)	7 (9.9%)	0 (0%)	2 (4.8%)	5 (10.2%)	10 (3.9%)	112 (8.7%)	
2	211 (31.9%)	37 (23.4%)	1 (1.4%)	3 (7.0%)	1 (2.4%)	14 (28.6%)	26 (10.0%)	293 (22.8%)	
3	283 (42.8%)	59 (37.3%)	11 (15.5%)	4 (9.3%)	10 (23.8%)	26 (53.1%)	63 (24.3%)	456 (35.5%)	
4	75 (11.3%)	15 (9.5%)	3 (4.2%)	24 (55.8%)	25 (59.5%)	4 (8.2%)	86 (33.2%)	232 (18.1%)	
Missing	34 (5.1%)	17 (10.8%)	49 (69.0%)	12 (27.9%)	4 (9.5%)	0 (0%)	74 (28.6%)	190 (14.8%)	
Lymph node involvement: N1									<0.001
No	391 (59.2%)	101 (63.9%)	66 (93.0%)	42 (97.7%)	37 (88.1%)	30 (61.2%)	212 (81.9%)	879 (68.5%)	
Yes	270 (40.8%)	57 (36.1%)	5 (7.0%)	1 (2.3%)	5 (11.9%)	19 (38.8%)	47 (18.1%)	404 (31.5%)	
Distant metastasis: M1									<0.001
No	547 (82.8%)	86 (54.4%)	69 (97.2%)	40 (93.0%)	31 (73.8%)	43 (87.8%)	187 (72.2%)	1003 (78.2%)	
Yes	114 (17.2%)	72 (45.6%)	2 (2.8%)	3 (7.0%)	11 (26.2%)	6 (12.2%)	72 (27.8%)	280 (21.8%)	
Margin status									<0.001
R0 Resection	354 (53.6%)	108 (68.4%)	35 (49.3%)	21 (48.8%)	31 (73.8%)	41 (83.7%)	191 (73.7%)	781 (60.9%)	
R1/R2 Resection	168 (25.4%)	26 (16.5%)	5 (7.0%)	18 (41.9%)	7 (16.7%)	7 (14.3%)	40 (15.4%)	271 (21.1%)	
Missing	139 (21.0%)	24 (15.2%)	31 (43.7%)	4 (9.3%)	4 (9.5%)	1 (2.0%)	28 (10.8%)	231 (18.0%)	
Neoadjuvant chemotherapy									<0.001
Yes	201 (30.4%)	30 (19.0%)	3 (4.2%)	5 (11.6%)	14 (33.3%)	3 (6.1%)	56 (21.6%)	312 (24.3%)	
No	455 (68.8%)	125 (79.1%)	68 (95.8%)	37 (86.0%)	27 (64.3%)	46 (93.9%)	201 (77.6%)	959 (74.7%)	
Missing	5 (0.8%)	3 (1.9%)	0 (0%)	1 (2.3%)	1 (2.4%)	0 (0%)	2 (0.8%)	12 (0.9%)	
Adjuvant chemotherapy									<0.001
Yes	335 (50.7%)	39 (24.7%)	7 (9.9%)	8 (18.6%)	23 (54.8%)	30 (61.2%)	102 (39.4%)	544 (42.4%)	
No	159 (24.1%)	91 (57.6%)	47 (66.2%)	32 (74.4%)	16 (38.1%)	17 (34.7%)	136 (52.5%)	498 (38.8%)	
Missing	167 (25.3%)	28 (17.7%)	17 (23.9%)	3 (7.0%)	3 (7.1%)	2 (4.1%)	21 (8.1%)	241 (18.8%)	
Neoadjuvant radiotherapy									
Yes	11 (1.7%)	5 (3.2%)	1 (1.4%)	7 (16.3%)	1 (2.4%)	0 (0%)	3 (1.2%)	28 (2.2%)	
No	635 (96.1%)	149 (94.3%)	70 (98.6%)	35 (81.4%)	41 (97.6%)	49 (100%)	250 (96.5%)	1229 (95.8%)	
Missing	15 (2.3%)	4 (2.5%)	0 (0%)	1 (2.3%)	0 (0%)	0 (0%)	6 (2.3%)	26 (2.0%)	
Adjuvant radiotherapy									<0.001
Yes	25 (3.8%)	4 (2.5%)	1 (1.4%)	8 (18.6%)	3 (7.1%)	1 (2.0%)	11 (4.2%)	53 (4.1%)	
No	479 (72.5%)	130 (82.3%)	53 (74.6%)	34 (79.1%)	39 (92.9%)	47 (95.9%)	231 (89.2%)	1013 (79.0%)	
Missing	157 (23.8%)	24 (15.2%)	17 (23.9%)	1 (2.3%)	0 (0%)	1 (2.0%)	17 (6.6%)	217 (16.9%)	
Type of resection									<0.001
Total pancreatectomy	71 (10.7%)	12 (7.6%)	19 (26.8%)	3 (7.0%)	2 (4.8%)	2 (4.1%)	14 (5.4%)	123 (9.6%)	
Distal pancreatectomy	435 (65.8%)	110 (69.6%)	43 (60.6%)	32 (74.4%)	30 (71.4%)	1 (2.0%)	125 (48.3%)	776 (60.5%)	
Pancreaticoduodenectomy	155 (23.4%)	36 (22.8%)	9 (12.7%)	8 (18.6%)	10 (23.8%)	46 (93.9%)	120 (46.3%)	384 (29.9%)	
Duration of surgery (minutes)									<0.001
Mean (SD)	315 (121)	313 (125)	302 (112)	389 (120)	321 (113)	338 (160)	335 (154)	300 (132)	
Missing	52 (7.9%)	21 (13.3%)	5 (7.0%)	2 (4.6%)	0 (0%)	4 (8.2%)	35 (13.5%)	118 (9.2%)	
Surgical access									<0.001
minimal-invasive	67 (10.1%)	20 (12.7%)	17 (23.9%)	0 (0%)	0 (0%)	0 (0%)	10 (3.9%)	114 (8.9%)	
Open surgery	594 (89.9%)	138 (87.3%)	54 (76.1%)	43 (100%)	42 (100%)	49 (100%)	249 (96.1%)	1169 (91.1%)	
Resected organs									0.005
Colon	210 (31.8%)	31 (19.6%)	16 (22.5%)	100 (38.6%)	29 (67.4%)	6 (14.3%)	5 (10.2%)	397 (30.9%)	
Stomach	197 (29.8%)	30 (19.0%)	19 (26.8%)	58 (22.4%)	8 (18.6%)	38 (90.5%)	4 (8.2%)	354 (27.6%)	
Adrenal gland	201 (30.4%)	30 (19.0%)	20 (28.2%)	46 (17.8%)	27 (62.8%)	7 (16.7%)	0 (0%)	331 (25.8%)	
Liver	120 (18.2%)	73 (46.2%)	3 (4.2%)	67 (25.9%)	1 (2.3%)	3 (7.1%)	18 (36.7%)	285 (22.2%)	
Kidney	78 (11.8%)	8 (5.1%)	2 (2.8%)	46 (17.8%)	25 (58.1%)	1 (2.4%)	0 (0%)	160 (12.5%)	
Small intestine	61 (9.2%)	18 (11.4%)	7 (9.9%)	24 (9.3%)	5 (11.6%)	3 (7.1%)	4 (8.2%)	122 (9.5%)	
Spleen	269 (40.7%)	67 (42.4%)	33 (46.5%)	86 (33.2%)	24 (55.8%)	18 (42.9%)	3 (6.1%)	500 (39.0%)	
Number of resected organs									0.115
1	343 (51.9%)	88 (55.7%)	41 (57.7%)	134 (51.7%)	6 (14.0%)	14 (33.3%)	23 (46.9%)	649 (50.6%)	
2	199 (30.1%)	44 (27.8%)	25 (35.2%)	66 (25.5%)	9 (20.9%)	16 (38.1%)	4 (8.2%)	363 (28.3%)	
3	77 (11.6%)	17 (10.8%)	3 (4.2%)	34 (13.1%)	15 (34.9%)	7 (16.7%)	1 (2.0%)	154 (12.0%)	
4 or more	38 (5.7%)	7 (4.4%)	0 (0%)	14 (5.4%)	12 (27.9%)	2 (4.8%)	0 (0%)	73 (5.7%)	
Missing	4 (0.6%)	2 (1.3%)	2 (2.8%)	11 (4.2%)	1 (2.3%)	3 (7.1%)	21 (42.9%)	44 (3.4%)	

^a^ Included leiomyosarcoma (*N* = 11), hepatocellular carcinoma (HCC; *N* = 13), gastrointestinal stromal tumors (GISTs; *N* = 22), and non-pancreatic neuroendocrine tumors (NET; *N* = 11)

The mean intraoperative blood loss for the overall cohort was 804 ml (SD: 1040). Missing data on blood loss ranged from 30.2 to 65.3% across groups (Table 2). Intraoperative adverse events occurred in 23.8% of patients. The highest rates were observed in liposarcoma (39.5%) and gastric adenocarcinoma (33.3%; *P* < 0.001). Reoperations were necessary in 16.6% of cases overall. Clinically relevant postoperative pancreatic fistulas (ISGPS grade B/C) were seen in 30.9% of patients. The highest rate was reported in gastric adenocarcinoma (45.2%) and the lowest in cystic pancreatic lesions (23.9%; *P* = 0.009). Delayed gastric emptying was observed in 16.1% of the cohort. Complications were further classified using the Clavien–Dindo classification. Grade III–V complications were recorded in 34.4% of patients. The highest rates were found in gastric adenocarcinoma (52.4%) whereas pancreatic NETs had the lowest rate (27.8%; *P* = 0.004). The mean ICU stay for the overall cohort was 3.7 days (SD: 9.1). The overall 90-day mortality rate was 6.9%. Gastric adenocarcinoma had the highest mortality rate (16.7%), followed by other malignancies (9.7%) and liposarcoma (7.0%). No deaths were recorded among patients with cystic pancreatic lesions.

**Table 2:** Outcomes for the overall cohort stratified by tumor entity.

	Pancreatic adenocarcinoma (*N* = 661)	Pancreatic NET (*N* = 158)	Cystic Pancreatic Lesions (*N* = 71)	Liposarcoma (*N* = 43)	Gastric adenocarcinoma (*N* = 42)	Cholangiocarcinoma (*N* = 49)	Other (*N* = 259)	Overall (*N* = 1283)	*P*-Value
Blood loss (in ml)									0.540
Mean (SD)	794 (978)	724 (933)	818 (971)	848 (1700)	513 (314)	716 (546)	931 (1260)	804 (1040)	
Missing	251 (38.0%)	70 (44.3%)	22 (31.0%)	13 (30.2%)	14 (33.3%)	32 (65.3%)	115 (44.4%)	517 (40.3%)	
Intraoperativeadverse events									<0.001
No	522 (79.0%)	132 (83.5%)	56 (78.9%)	26 (60.5%)	28 (66.7%)	36 (73.5%)	178 (68.7%)	978 (76.2%)	
Yes	139 (21.0%)	26 (16.5%)	15 (21.1%)	17 (39.5%)	14 (33.3%)	13 (26.5%)	81 (31.3%)	305 (23.8%)	
Reoperation									0.075
Yes	101 (15.3%)	19 (12.0%)	10 (14.1%)	7 (16.3%)	11 (26.2%)	11 (22.4%)	54 (20.8%)	213 (16.6%)	
No	538 (81.4%)	135 (85.4%)	61 (85.9%)	33 (76.7%)	29 (69.0%)	36 (73.5%)	197 (76.1%)	1029 (80.2%)	
Missing	22 (3.3%)	4 (2.5%)	0 (0%)	3 (7.0%)	2 (4.8%)	2 (4.1%)	8 (3.1%)	41 (3.2%)	
Bleeding									0.135
No	573 (86.7%)	143 (90.5%)	63 (88.7%)	37 (86.0%)	37 (88.1%)	41 (83.7%)	209 (80.7%)	1103 (86.0%)	
Yes	88 (13.3%)	15 (9.5%)	8 (11.3%)	6 (14.0%)	5 (11.9%)	8 (16.3%)	50 (19.3%)	180 (14.0%)	
Pancreatic fistula									0.009
No	478 (72.3%)	110 (69.6%)	54 (76.1%)	27 (62.8%)	23 (54.8%)	35 (71.4%)	159 (61.4%)	886 (69.1%)	
Yes	183 (27.7%)	48 (30.4%)	17 (23.9%)	16 (37.2%)	19 (45.2%)	14 (28.6%)	100 (38.6%)	397 (30.9%)	
DGE									0.019
No	546 (82.6%)	148 (93.7%)	62 (87.3%)	33 (76.7%)	35 (83.3%)	40 (81.6%)	212 (81.9%)	1076 (83.9%)	
Yes	115 (17.4%)	10 (6.3%)	9 (12.7%)	10 (23.3%)	7 (16.7%)	9 (18.4%)	47 (18.1%)	207 (16.1%)	
CD-classification									0.004
none	184 (27.8%)	44 (27.8%)	29 (40.8%)	7 (16.3%)	9 (21.4%)	9 (18.4%)	52 (20.1%)	334 (26.0%)	
Grade I-II	261 (39.5%)	70 (44.3%)	20 (28.2%)	20 (46.5%)	11 (26.2%)	22 (44.9%)	104 (40.2%)	508 (39.6%)	
Grade III-V	216 (32.7%)	44 (27.8%)	22 (31.0%)	16 (37.2%)	22 (52.4%)	18 (36.7%)	103 (39.8%)	441 (34.4%)	
Duration of ICU stay (days)									0.058
Mean (SD)	3.29 (6.25)	2.77 (7.55)	1.98 (4.08)	3.66 (4.97)	5.88 (8.32)	4.68 (6.59)	5.02 (14.8)	3.73 (9.13)	
Missing	156 (23.6%)	25 (15.8%)	19 (26.8%)	1 (2.3%)	1 (2.4%)	9 (18.4%)	15 (5.8%)	226 (17.6%)	
Mortality (90-day)									0.008
Yes	44 (6.7%)	7 (4.4%)	0 (0%)	3 (7.0%)	7 (16.7%)	3 (6.1%)	25 (9.7%)	89 (6.9%)	
No	583 (88.2%)	148 (93.7%)	68 (95.8%)	40 (93.0%)	32 (76.2%)	40 (81.6%)	210 (81.1%)	1121 (87.4%)	

^a^ Included leiomyosarcoma (*N* = 11), hepatocellular carcinoma (HCC; *N* = 13), gastrointestinal stromal tumors (GISTs; *N* = 22), and non-pancreatic neuroendocrine tumors (NET; *N* = 11)

In the univariable ordinal regression analysis (Table 3), several patient- and surgery-related factors were significantly associated with the occurrence of postoperative complications. Among demographic variables, female gender was associated with a reduced risk of complications compared to males (OR: 0.79, 95% CI: 0.64–0.96, *P* = 0.020), while age and BMI had no significant impact.

**Table 3:** Univariable ordinal regression for postoperative complications.

	Complications		
***Predictors***	***OR***	***CI***	***P***
Age (in years)	1.00	0.99–1.01	0.988
Gender (Ref: Male)			
Female	0.79	0.64–0.96	0.020
CCI (Points)	1.24	1.05–1.46	0.010
BMI (kg/m^2^)	1.01	0.96–1.06	0.657
ASA (Ref: ASA 1)			
ASA 2	2.39	1.76–3.25	<0.001
ASA 3	2.90	2.11–3.99	<0.001
ASA 4	8.36	3.54–21.37	<0.001
Tumor entity (Ref: Pancreatic adenocarcinoma)			
Cholangiocarcinoma	1.40	0.83–2.38	0.206
Gastric adenocarcinoma	2.09	1.15–3.87	<0.001
Pancreatic NET	0.88	0.64–1.21	0.434
Cystic pancreatic lesions	0.66	0.41–1.05	0.082
Liposarcoma	1.26	0.73–2.20	0.403
Other	1.42	1.09–1.85	0.008
Neoadjudjuvant chemotherapy (Ref: Yes)			
No	1.05	0.83–1.32	0.677
Neoadjuvant radiotherapy (Ref: Yes)			
No	1.33	0.68–2.63	0.404
Type of resection (Ref: Total pancreatectomy)			
Distal pancreatectomy	0.83	0.59–1.17	0.289
Pancreatoduodenectomy	1.32	0.91–1.91	0.139
Type of surgical access (Ref: Minimal invasive surgery)			
Open surgery	3.05	2.15–4.34	<0.001
Duration of surgery (Ref: Short)			
Long	1.66	1.32–2.09	<0.001
Case load (Ref: less than 50 cases)			
More than 50 cases	0.68	0.55–0.85	<0.001

Higher comorbidity burden, increased the odds of complications (OR: 1.24, 95% CI: 1.05–1.46, *P* = 0.01). Similarly, increasing ASA scores were strongly associated with postoperative morbidity. Patients with gastric adenocarcinoma had significantly higher risks of complications compared to those with pancreatic adenocarcinoma (OR: 2.09, 95% CI: 1.15–3.87, *P* < 0.001), while patients with cystic pancreatic lesions showed a non-significant trend toward fewer complications (OR: 0.66, *P* = 0.082). Neoadjuvant treatments were not associated with postoperative outcomes. Open surgical access was significantly associated with higher complication risk compared to minimally invasive surgery (OR: 3.05, 95% CI: 2.15–4.34, *P* < 0.001), as was longer duration of surgery (OR: 1.66, 95% CI: 1.32–2.09, *P* < 0.001). Duration of surgery was defined as follows: short (<240 minutes) vs long (≥240 minutes). Higher institutional case load (more than 50 cases) appeared to be protective (OR: 0.68, 95% CI: 0.55–0.85, *P* < 0.001).

Focusing on patients with pancreatic tumors (Table 4), multivisceral resection involving the colon (OR: 1.78, *P* < 0.001) and stomach (OR: 1.33, *P* = 0.042) was significantly associated with an increased risk of postoperative complications. Regarding the extent of surgery, resection of three organs was significantly associated with increased complications (OR: 1.75, *P* = 0.006).

**Table 4:** Univariable ordinal regression for postoperative complications for patients with pancreatic entity including pancreatic adenocarcinoma, pancreatic NET and cystic pancreatic lesions.

	Complications		
***Predictors***	***OR***	***CI***	***P***
Colon (Ref: No)	1.78	1.36–2.33	<0.001
Stomach (Ref: No)	1.33	1.01–1.74	0.042
Adrenal gland (Ref: No)	0.95	0.72–1.24	0.695
Liver (Ref: No)	1.08	0.81–1.44	0.598
Kidney (Ref: No)	1.11	0.74–1.65	0.622
Small intestine (Ref: No)	1.41	0.95–2.09	0.087
Spleen (Ref: No)	0.75	0.58–0.96	0.022
Number of resected organs (ref: 1)			
2	1.28	0.97–1.69	0.085
3	1.75	1.17–2.63	0.006
4 or more	1.54	0.90–2.63	0.116

In the final multivariable ordinal regression model (Table 5), three independent predictors of postoperative complications remained significant: ASA classification, duration of surgery, and type of surgical access. Longer operations remained a significant risk factor (OR: 1.65, 95% CI: 1.29–2.10, *P* < 0.001), and open surgery was associated with a twofold increase in complication risk (OR: 2.10, 95% CI: 1.40–3.17, *P* < 0.001).

**Table 5:** Final multivariable ordinal regression model for postoperative complications using backward selection approach.

	Complications		
***Predictors***	***Odds Ratios***	***CI***	***P***
ASA (Ref: ASA 1)			
ASA 2	1.89	1.31–2.72	0.001
ASA 3	2.19	1.51–3.18	<0.001
ASA 4	6.83	2.81–17.92	<0.001
Duration of surgery (Ref: Short)			
Long	1.65	1.29–2.10	<0.001
Type of surgical access (Ref: Minimal-invasive surgery)			
Open surgery	2.10	1.40–3.17	<0.001

In the univariable logistic regression analysis (Table 6), several factors demonstrated significant associations with 90-day mortality. Female gender was associated with significantly lower risk of mortality compared to males (OR: 0.55, 95% CI: 0.35–0.86, *P* = 0.009). Increasing comorbidity burden, as measured by the CCI, significantly increased the risk of mortality (OR: 1.30, 95% CI: 1.11–1.52, *P* = 0.001). Among ASA classes, only ASA 4 was significantly associated with increased mortality (OR: 4.97, 95% CI: 1.43–15.62, *P* = 0.007).

**Table 6:** Univariable logistic regression for mortality.

	Mortality (90-day)		
***Predictors***	***OR***	***CI***	***P***
Age (in years)	1.00	0.99–1.01	0.834
Gender (Ref: Male)			
Female	0.55	0.35–0.86	0.009
CCI (Points)	.30	1.11–1.52	0.001
BMI (kg/m^2)	1.00	0.98–1.01	0.955
ASA (Ref: ASA 1)			
ASA 2	0.93	0.47–1.98	0.850
ASA 3	1.63	0.84–3.42	0.165
ASA 4	4.97	1.43–15.62	0.007
Tumor entity (Ref: Pancreatic adenocarcinoma)			
Cholangiocarcinoma	0.99	0.23–2.88	0.992
Gastric adenocarcinoma	2.90	1.12–6.60	0.017
Pancreatic NET	0.63	0.25–1.33	0.263
Cystic Pancreatic Lesions	0.02	0.00–6.93	0.975
Liposarcoma	0.99	0.23–2.88	0.992
Other	1.58	0.93–2.62	0.083
Neoadjuvant chemotherapy (Ref: Yes)			
No	1.08	0.66–1.86	0.758
Neoadjuvant radiotherapy (Ref: Yes)			
No	1.03	0.30–6.46	0.968
Type of resection (Ref: Total pancreatectomy)			
Distal pancreatectomy	0.95	0.46–2.21	0.893
Pancreatoduodenectomy	1.33	0.62–3.17	0.493
Type of surgical access (Ref: Minimal-invasive surgery)			
Open surgery	5.16	1.61–31.59	0.023
Duration of surgery (Ref: Long)			
Short	1.78	1.06–3.17	0.039
Case load (Ref: less than 50 cases)			
More than 50 cases	0.61	0.40–0.96	0.030

Tumor type also influenced outcomes. Patients with gastric adenocarcinoma had higher odds of mortality compared to those with pancreatic adenocarcinoma (OR: 2.90, 95% CI: 1.12–6.60, *P* = 0.017).

Open surgical access was associated with a markedly increased risk of mortality (OR: 5.16, 95% CI: 1.61–31.59, *P* = 0.023) compared to minimally invasive approaches. Interestingly, short duration of surgery was associated with increased risk of death (OR: 1.78, 95% CI: 1.06–3.17, *P* = 0.039). High institutional case volume (>50 cases) was protective (OR: 0.61, 95% CI: 0.40–0.96, *P* = 0.030).

Subgroup analysis for patients with pancreatic tumors (Table 7) did not identify any statistically significant association between multivisceral resection and 90-day mortality, though resection involving the colon (OR: 1.68, *P* = 0.082) or liver (OR: 1.70, *P* = 0.090) showed non-significant trends toward higher mortality.

**Table 7:** Univariable logistic regression for mortality for patients with pancreatic entity including pancreatic adenocarcinoma, pancreatic NET and cystic pancreatic lesions.

	Mortality (90-day)		
***Predictors***	***OR***	***CI***	***P***
Colon (Ref: No)	1.68	0.92–2.98	0.082
Stomach (Ref: No)	0.79	0.39–1.50	0.498
Adrenal gland (Ref: No)	0.68	0.33–1.30	0.270
Liver (Ref: No)	1.70	0.90–3.10	0.090
Kidney (Ref: No)	0.74	0.22–1.89	0.580
Small intestine (Ref: No)	1.25	0.47–2.82	0.620
Spleen (Ref: No)	1.00	0.56–1.77	1.000
Number of resected organs (ref: 1)			
2	1.36	0.71–2.54	0.346
3	1.53	0.59–3.50	0.341
4 or more	0.85	0.13–3.01	0.833

The final multivariable logistic regression model (Table 8), derived using a backward selection approach, identified three independent predictors of 90-day mortality: gender, ASA classification, and duration of surgery. Female gender remained protective (OR: 0.46, 95% CI: 0.27–0.77, *P* = 0.004). ASA 4 emerged as a strong independent risk factor, with a nine fold increase in mortality compared to ASA 1 (OR: 9.02, 95% CI: 2.22–37.08, *P* = 0.002). Finally, shorter duration of surgery was paradoxically associated with higher mortality (OR: 2.34, 95% CI: 1.28–4.62, *P* = 0.009). The type of pancreatic resection was not an independent predictor of morbidity or mortality.

**Table 8:** Final multivariable logistic regression model for mortality using backward selection approach.

	Mortality (90-day)^a^		
***Predictors***	***Odds Ratios***	***CI***	***P***
Gender (Ref: Male)			
Female	0.46	0.27–0.77	0.004
ASA (Ref: ASA 1)			
ASA 2	1.28	0.52–3.86	0.618
ASA 3	2.27	0.95–6.75	0.094
ASA 4	9.02	2.22–37.08	0.002
Duration of surgery (Ref: Long)			
Short	2.34	1.28–4.62	0.009

## Discussion

This multicenter study represents the largest cohort to date evaluating multivisceral pancreatic resections across a wide spectrum of malignancies. We observed a 90-day mortality rate of 6.9%, with severe complications occurring in 34.4% of cases. While pancreatic NET patients tended to be younger and exhibited the lowest rates of major complications and 90-day mortality, patients with gastric adenocarcinoma and liposarcoma experienced the highest surgical morbidity and mortality. The increased morbidity and mortality in non-pancreas-associated indications might be attributable to the fact that these procedures were not primarily performed by dedicated pancreatic surgeons, potentially reflecting limited experience. Multivariable analysis identified key predictors of adverse outcomes: higher ASA classification and open surgical approaches were independently associated with both increased morbidity and mortality, while prolonged operative time was an independent predictor of morbidity alone. Notably, higher institutional case volume appeared to be protective, reinforcing the importance of experience and specialization in achieving optimal outcomes in these complex procedures.

The large published meta-analysis and systematic review on this topic analyzed three studies with a total of 176 patients undergoing multivisceral pancreatic resections for pancreatic ductal adenocarcinoma (PDAC) compared to 713 standard pancreatectomy patients^[8]^. In our analysis 661 patients had PDAC. In the review by Petrucciani and colleagues, postoperative morbidity was higher in patients undergoing multivisceral resections (56–69%) compared to standard pancreatectomy (37–50%), with in-hospital mortality reaching 10% in multivisceral resections versus 4% in standard pancreatectomy. Similarly, our study found a severe complication rate of 32.7% and a 90-day mortality rate of 6.7% on PDAC patients. The largest prior series on PDAC multivisceral resections included 101 patients.^[1]^ While both that study and ours demonstrate feasibility, our international, register-based analysis includes a larger, more diverse cohort and examines outcomes across multiple tumor types. Unlike the prior comparison with standard resections, we identified risk factors and survival predictors. Their reported 90-day mortality was 3.0%, compared to 6.7% in our study – likely due to higher case complexity and center variability. Severe complications occurred in 32.0%, with higher ASA scores, longer surgeries, and open approaches increasing morbidity.

In another case series Burdelski *et al*^[11]^ analyzed 55 patients with multivisceral pancreatic resections comparing them to 154 palliative bypass and 303 standard pancreatic head resections. This study identified kidney resection and intraoperative transfusion as independent risk factors, whereas our study highlighted higher ASA classification, prolonged operative time, and open surgery as key predictors of morbidity and mortality.

In a study from the China National Cancer Center 210 patients with pT4b gastric cancer underwent multivisceral resection, with a hospital mortality rate of 0.5% and a complication rate of 8.1%^[2]^ The most common postoperative complications were anastomotic leaks (4.3%) and intra-abdominal infections (5.7%). Another study that compared 64 multivisceral resection patients to 621 standard gastrectomy patients, showed that multivisceral resections were associated with increased morbidity, higher 90-day mortality, and more advanced disease^[7]^. However, after propensity matched score, these differences disappeared, suggesting that multivisceral resections do not inherently increase surgical risk when patients are carefully selected. In contrast, our study focused on gastric cancer patients undergoing multivisceral resections involving the pancreas, revealing a higher 90-day mortality rate (16.7%) and severe complications in 53.7% of cases, indicating that pancreatic involvement may substantially increase surgical risk.

Both our study and a study by Hilal *et al*^[12]^ on multivisceral pancreatic resections for NETs highlight the potential benefits of aggressive surgical approaches for locally advanced NETs. However, the two studies differ in cohort size, surgical outcomes, and survival results. The study from Italy analyzed only 12 patients undergoing multivisceral resection for NETs, with a median tumor size of 9.5 cm and a median of three additional organs resected. Notably, there were no postoperative deaths, and while complications occurred in five patients (major in three), median disease-free survival was not reached, suggesting that NET patients may benefit from aggressive resections with relatively low morbidity and long-term survival. In contrast, our study examined a much larger cohort including a significant subgroup of NET patients. In our study, NET patients who underwent multivisceral resection involving the pancreas had a lower overall complication rate (27.8%) compared to other tumor types, and the lowest 90-day mortality rate among the analyzed malignancies. The R0 resection rate in our study (68.5%), was high. This aligns with the findings from a study by Abu Hilal, reinforcing the idea that multivisceral resection in NETs can provide good local tumor clearance. Although favorable short-term outcomes were observed in NET patients, no conclusions regarding long-term benefit can be drawn, particularly in metastatic disease.

An Italian study analyzed 59 patients who underwent distal pancreatectomy with multivisceral resections. Similar to our findings, this demonstrated that multivisceral resections, which involve increased operative complexity, were associated with worse outcomes. In the cohort study by Panzeri, these procedures led to longer operative times (291 vs 227 minutes, *P* < 0.001) and a higher need for intraoperative transfusions (21.4% vs 3.3%, *P* < 0.001)^[13]^.

This study has several limitations. First, its retrospective, register-based design introduces inherent selection biases and limits the ability to establish causal relationships between surgical factors and outcomes. Additionally, despite including data from multiple high-volume centers, variations in surgical techniques, perioperative management, and institutional protocols may have influenced the results. Approximately 10% of the data originated from a single center, potentially introducing center-specific bias; however, total case volumes per center were not captured by the registry. Variations in institutional experience and case volume may have influenced perioperative outcomes and could partly explain the association between shorter operative times and increased mortality. Another limitation is the heterogeneity of tumor entities included in the cohort, which, while reflective of real-world practice, may introduce confounding factors when comparing outcomes across different malignancies. Although our findings suggest that open surgical access is significantly associated with a higher risk of postoperative complications compared to minimally invasive surgery, this result may be influenced by selection bias inherent in retrospective analyses. It is likely that minimally invasive procedures were more frequently performed in fitter patients undergoing less extensive and technically simpler resections. Due to the small proportion of minimally invasive cases, the heterogeneity of the cohort, and the multicenter nature of the study, additional subgroup analyses or propensity score matching were not feasible. Consequently, the results regarding minimally invasive approaches should be interpreted cautiously.

Interestingly, shorter operative time emerged as an independent predictor of 90-day mortality, a finding that is somewhat counterintuitive and warrants further discussion. This association most likely reflects selection bias rather than a causal effect, as critically ill patients or those with unfavorable intraoperative findings often underwent reduced or abbreviated procedures, resulting in shorter operative times but higher mortality. Furthermore, data on long-term functional outcomes and quality of life after multivisceral resection were not analyzed, limiting our understanding of the broader impact of these procedures beyond survival. Furthermore, R1 and R2 resections were collected as a combined variable in the registry, which limits interpretation of the oncologic impact of margin status. Although patients with known metastases were excluded, the relatively high R1/R2 rate suggests that some occult metastatic or locally advanced disease may have been present.

A relatively high proportion of missing data was observed for certain variables, including intraoperative blood loss, resection margin status, and ICU stay. As these parameters were analyzed using a complete case approach, the potential for bias cannot be excluded and should be considered when interpreting the results. Most missing values in our dataset occurred in categorical variables. Since classical imputation methods are not applicable in this context and introducing an artificial “missing” category is not clinically meaningful, these variables were analyzed as reported, which should be considered when interpreting the descriptive statistics. The cause of the higher mortality rate observed in patients undergoing multivisceral resections for gastric adenocarcinoma remains speculative due to a lack of center-specific data and should be interpreted with caution. The potential contribution of non-pancreatic specialist surgery to higher mortality should be regarded as conjectural and requires confirmation in future studies with more detailed datasets.

Finally, for the purpose of this study, we focused on multivisceral resections involving additional organ removal beyond the standard anatomical components of pancreatoduodenectomy or distal pancreatectomy. This approach was chosen to specifically evaluate the risks and outcomes associated with extended resections. While necessary for consistency across centers, this definition may limit comparability with studies applying broader definitions of multivisceral surgery. Future research will focus on further subgroup analyses to better define patient-specific risk profiles and identify factors influencing postoperative morbidity, mortality, and survival outcomes. The role of minimally invasive and robotic-assisted techniques in reducing perioperative morbidity and improving survival should also be explored further. Finally, advanced survival analyses integrating clinical, imaging, and molecular biomarkers could aid in refining risk stratification models, ultimately improving individualized treatment strategies for patients undergoing multivisceral oncologic resections involving the pancreas.

## Conclusions

Multivisceral pancreatic resections are feasible with acceptable outcomes in selected patients. Higher ASA scores and open surgery were independently associated with increased morbidity and mortality, while female gender and high institutional case volume were protective. Among patients with pancreatic tumors, multivisceral resections involving the colon or stomach significantly increased the risk of postoperative complications, while the resection of exactly three organs was associated with a higher complication rate.

## Data Availability

The datasets generated and/or analyzed during the current study are not publicly available due to local data protection regulations but are available from the corresponding author upon reasonable request and subject to institutional approval.

## Research registration unique identifying number (UIN)

1. Registry used: https://www.clinicaltrials.gov/. 2. Unique Identifying number or registration ID: NCT07091097. 3. Hyperlink: https://clinicaltrials.gov/study/NCT07091097.
